# Measurement equivalence of the Four-Dimensional Symptom Questionnaire (4DSQ) in adolescents and emerging adults

**DOI:** 10.1371/journal.pone.0221904

**Published:** 2019-08-29

**Authors:** Berend Terluin, Johannes C. van der Wouden, Henrica C. W. de Vet

**Affiliations:** 1 Department of General Practice and Elderly Care Medicine, Amsterdam Public Health research institute, Amsterdam UMC–Vrije Universiteit Amsterdam, Amsterdam, The Netherlands; 2 Department of Epidemiology and Biostatistics, Amsterdam Public Health research institute, Amsterdam UMC–Vrije Universiteit Amsterdam, Amsterdam, The Netherlands; Fordham University, UNITED STATES

## Abstract

The Four-Dimensional Symptom Questionnaire (4DSQ) is a self-report instrument measuring distress, depression, anxiety and somatization. The questionnaire has been developed and validated in adult samples. It is unknown whether adolescents and emerging adults respond to the 4DSQ items in the same way as adults do. The objective of the study was to examine measurement equivalence of the 4DSQ across adolescents, emerging adults and adults. 4DSQ data were collected in a primary care psychotherapy practice (N = 1349). Measurement equivalence was assessed using differential item and test functioning (DIF and DTF) analysis in an item response theory framework. DIF was compared across the following groups: adolescents (age 10–17), emerging adults (age 18–25), and adults (age 26–40). DIF was found in 9 items (out of 50) across adolescents and adults, and in 4 items across emerging adults and adults. The item with the largest DIF was ‘difficulty getting to sleep’, which was less severe for adolescents compared to adults. A likely explanation is that adolescents have a high base rate for problems with sleep initiation. The effect of DIF on the scale scores (DTF) was negligible. Adolescents and emerging adults score some 4DSQ items differently compared to adults but this had practically no effect on 4DSQ scale scores. 4DSQ scale scores from adolescents and emerging adults can be interpreted in the same way as 4DSQ scores from adults.

## Introduction

Adolescence and ‘emerging adulthood’ represent two consecutive transitional periods between childhood and full-fledged adulthood. Adolescence, roughly spanning ages 10–17, is characterized by physical maturation under the influence of sex hormones, and by psychological and social changes [1]. Emerging adulthood, starting at age 18 and ending in the mid-twenties, is characterized by identity explorations in love, work and worldview in preparation for the roles and responsibilities of adulthood [2]. Both periods come with an unmistakable vulnerability to the development of mental disorders. Between age 11 and age 21 the prevalence of mental disorders doubles up to about 40% [3]. Thereafter, the prevalence gradually drops again to 24% at age 26, and 17% at age 30 [4]. The fact that about three-fourth of all lifetime mental disorders have their onset before the mid-twenties [5] indicates the importance of adolescence and emerging adulthood for the mental health of the population.

The measurement of mental health symptoms in adolescents and emerging adults, using self-report questionnaires, may be challenging because they may not have developed the required ‘emotional competence’ enabling them to recognize their personal feelings and to use the vocabulary to communicate their emotions [6, 7]. Adolescents and emerging adults may not comprehend a questionnaire’s items in the same way as adults do. Consequently, the items of a questionnaire may (partly) tap different constructs in adolescents and emerging adults than they do in adults, causing the same questionnaire to measure (partly) different things. In that case, the questionnaire scores from adolescents and emerging adults may not be comparable with scores from adults. This is a major concern when a questionnaire that has been developed and validated in an adult population is used in adolescents and emerging adults.

The Four-Dimensional Symptom Questionnaire (4DSQ) is a self-report questionnaire measuring distress, depression, anxiety and somatization [8]. The 4DSQ was initially developed in adult family practice patients, to be applied later in occupational health care [9] and in outpatient mental health care [10]. Although the 4DSQ was developed and validated in adult populations, some primary care psychologists, involved in the treatment of adolescents and emerging adults, started to use the 4DSQ in these groups.

There are many mental health measures used in adolescents and/or emerging adults. A recent review identified 117 measures [11] whereas another review identified 29 instruments [12]. These instruments cover a variety of domains, including symptoms, social functioning, cognitions, emotions, quality of life, and others. The 4DSQ consists of 4 symptom scales, 3 of which cover relatively narrow concepts (depression, anxiety, somatization). The fourth scale, which covers a much broader concept (distress), actually indexes psychological suffering of any kind, irrespective of its specific cause. The 4DSQ supports the distinction between general distress and psychiatric disorder (in particular depression and anxiety) in adult persons [13], and hopefully will do so in adolescents and emerging adults too.

Therefore, we aimed to examine whether the 4DSQ measures the same constructs (i.e. distress, depression, anxiety and somatization) in adolescents and emerging adults in the same way as it does in adults. If it does, the 4DSQ is said to possess ‘measurement equivalence’ or ‘measurement invariance’ across adolescents, emerging adults and adults [14]. Our research hypothesis was that the 4DSQ was measurement equivalent across adolescents, emerging adults and adults.

## Materials and methods

### Participants and procedure

4DSQ data were collected in consecutive new clients of a primary care psychotherapy practice in the Netherlands, between December 2002 and February 2014. Primary care psychotherapy practices in the Netherlands constitute community-based outpatient clinics where people turn to for professional help for a variety of psychological and psychosocial problems, e.g., stress, burnout, emotional symptoms, bereavement, and mental problems pertaining to health, relationships, study/work and sexuality. People can be referred by their family physician or apply on their own initiative. Initially, the data were collected as part of the routine intake assessment of new clients. Only later, the idea was born to use the 4DSQ data for research. 4DSQ data were stored in a digital score manager, along with the clients’ age and gender. No other patient information was available. For the present study, the data were made available fully anonymized and de-identified. Ethical approval and informed consent were not required for this study (Medical Ethics Review Committee of the VU University Medical Center, Amsterdam, reference 2017.569 and 2018.257). We selected clients aged 10–17 as the adolescent group (focal group 1), clients aged 18–25 as the emerging adult group (focal group 2), and clients aged 26–40 as the adult group (reference group).

### Measure

The 4DSQ is a 50-item self-report questionnaire consisting of 4 separate scales for distress (16 items), depression (6 items), anxiety (12 items) and somatization (16 items). The items inquire about the frequency of symptoms, feelings and thoughts during the past week, offering a 5-point response scale from ‘no’ to ‘very often or constantly’. To calculate scale scores the item responses are scored on a 3-point ordinal scale: ‘no’ (= 0 points), ‘sometimes’ (= 1 point) and ‘regularly’, ‘often’ and ‘very often or constantly’ (= 2 points). The rationale behind collapsing ‘regularly’, ‘often’ and ‘very often or constantly’ into a single code is to minimize contamination of the scale scores by personality-related extreme response tendencies [15]. The 4DSQ is freely available for non-commercial use as in research and health care, and is available in several translations at www.4dsq.eu.

The distress scale measures the non-specific discomforting emotional state people experience in response to stressors, in particular when they feel they are not coping effectively [16]. An elevated distress score indicates non-specific psychological suffering, or in simple language, that the person is having a difficult time. Typical distress symptoms include irritability, feeling tense, worry, and sleeping problems. The distress score has shown to be associated with psychosocial difficulties and life events, job stress, (psycho-)social functioning and sickness absence [8]. In family practice patients, the distress score best predicts any psychosocial diagnosis established by the family physician [8]. Internal consistency reliability (Cronbach’s alpha) of the distress scale is > 0.90 across a range of populations [8].

The depression scale measures specific symptoms of depressive disorder, i.e. loss of pleasure (anhedonia) and negative cognitions [17, 18]. An elevated depression score indicates relatively increased probability of a moderate or severe DSM-IV major depressive disorder [19]. Cronbach’s alpha is > 0.80 across a range of populations [8].

The anxiety scale measures specific symptoms of anxiety disorders, i.e. pathological anxiety such as free floating and phobic anxiety [20]. An elevated anxiety score indicates relatively increased probability of one or more DSM-IV anxiety disorders [19, 21]. Cronbach’s alpha is > 0.80 across a range of populations [8].

The somatization scale measures a range of medically unexplained physical symptoms [22]. An elevated somatization score indicates that the respondent is suffering from somatoform symptoms and bodily distress [23, 24]. Several mechanisms can be in play including somatosensory amplification, illness attribution, illness behavior [25], and central sensitization [26]. The somatization score best predicts the family physician’s suspicion of a psychosocial background in patients presenting with physical complaints [8]. Cronbach’s alpha is > 0.80 across a range of populations [8].

### Statistical analysis

#### Measurement equivalence

A multi-item scale demonstrates measurement equivalence if the scale measures the same construct (e.g., distress) across different groups of respondents (e.g., age groups) and its items function the same as indicators of that construct across the groups [27]. The idea of items as indicators of an underlying construct means that the scoring of the items depend on the respondents’ standing on the construct. For instance, a respondent with a relatively low level of distress will probably not respond to most items of a distress scale and obtain a low score. On the other hand, a respondent with a high level of distress will probably respond to many items of the scale and obtain a much higher score. The key issue in multi-item scale measurement is the relationship between the items and the construct the items are purported to measure such that a certain level of the construct leads to certain responses to the items and to a certain total score. A multi-item scale is measurement equivalent across different groups only when the item–construct relationships are the same in those groups, meaning that the same level of the construct leads to the same score. There are many approaches to the analysis of item–construct relationships, which are collectively called ‘differential item functioning’ (DIF) analysis [27]. We chose DIF analysis within an item response theory (IRT) framework for 2 reasons. First, because IRT directly models the relationships between a construct and item responses [28, 29]. And second, because IRT provides an elegant approach to evaluate the impact of DIF on the scale score, called ‘differential test functioning’ (DTF) analysis [30]. After all, researchers and clinicians are not so much interested in item scores but more so in scale (or total) scores.

However, before using IRT for DIF and DTF analyses, we needed to ascertain whether the application of IRT was justified. A key assumption of IRT is that the scale is unidimensional. Therefore, our analyses involved three steps: (1) dimensionality analysis, (2) DIF analysis, and (3) DTF analysis. Each 4DSQ scale was analyzed separately.

#### Dimensionality

The application of IRT requires that scales only have one dimension (or factor) underlying its item responses. However, psychological scales rarely have just one underlying dimension [31, 32]. They typically include multiple dimensions that appear statistically as additional factors, apart from the primary factor of interest. A scale is considered ‘essentially unidimensional’ when its item responses are primarily driven by one large general factor, whereas additional smaller factors do not impact the scale scores too much [33]. A convenient way to assess whether a scale is essentially unidimensional (i.e. unidimensional enough to be treated as unidimensional) is by modeling the scale as a bifactor model [34–36]. A bifactor model is characterized by a large ‘general factor’ underlying the responses of all items, and a number of smaller ‘specific factors’ underlying subsets of items [37]. Relevant statistics of bifactor models are the proportion of uncontaminated correlations (PUC), the explained common variance (ECV), and omega-hierarchical (omega-h) [34]. The PUC is an index of how many inter-item correlations are accounted for by the general factor alone. The ECV is an index of the relative general factor strength, representing the proportion of the total common variance that is accounted for by the general factor. Omega-h represents the proportion of the total score variance that is accounted for by the general factor [38]. Indicative of essential unidimensionality are: PUC values greater than 0.8 [34], or ECV values greater than 0.6 [34, 35], or omega-h values greater than 0.8, or omega-h values between 0.7 and 0.8 combined with PUC values greater than 0.7 [34].

We used confirmatory factor analysis (CFA) within the framework of structural equation modelling [39] to examine bifactor models, using diagonally weighted least squares (DWLS) as estimator and mean and variance adjusted test statistics. The item responses were treated as ordered categories. Model fit was assessed by the following scaled fit indices: comparative fit index (CFI), Tucker-Lewis index (TLI), root mean square error of approximation (RMSEA), and standardized root mean squared residual (SRMR). The following criteria were taken as indicating adequate fit: CFI and TLI > 0.95, RMSEA < 0.06 and SRMR < 0.08 [40]. The analyses were performed scale-by-scale and in each age group separately. We first fitted a strictly unidimensional model (comprising only the general factor) and subsequently fitted a bifactor model by defining one or more specific factors based on the modification indices and the standardized expected parameter change [41]. The absence of local item dependence (LID), an indication that the measurement model was properly specified, was checked by inspecting residual correlations (absolute correlations > 0.2 were taken as evidence of LID).

#### Differential item functioning

**Fig 1:**
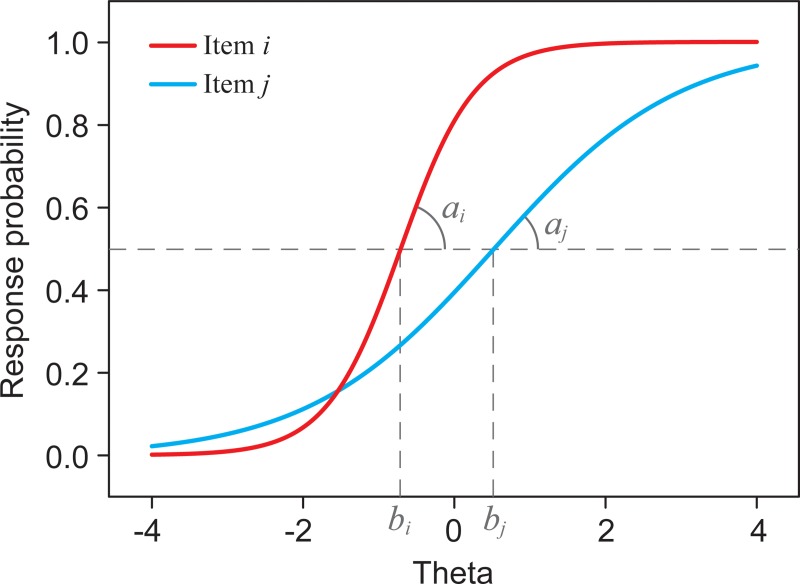
Item characteristic curves of 2 dichotomous items. Response probabilities as a function of the latent trait (theta) and the item characteristics ‘discrimination’ (*a*_*i*_ and *a*_*j*_) and ‘severity’ (*b*_*i*_ and *b*_*j*_).

Differential item functioning (DIF) means that persons from different groups (e.g. gender, age, education) have different probabilities of responding to an item, despite being on the same level of the construct being measured [42]. An item with DIF may systematically favor one group over the other, thus resulting in measurement bias, and jeopardizing the comparison between the groups. IRT directly models the probability of responding to an item as a function of the construct being measured, given certain item characteristics [28, 29]. The construct is treated as a latent variable (i.e. a latent trait) and the relationship between the item and the trait underlying that item is determined by 2 item characteristics: ‘severity’ and ‘discrimination’. Fig 1 presents 2 item characteristic curves, a graphical representation of the item-trait relationships of 2 dichotomous items *i* and *j*. The metric of the underlying trait (denoted ‘theta’) is presented on a standardized scale with a mean value of 0 and a standard deviation of 1. The item characteristic curves differ with respect to their slopes and to their location relative to the theta scale. The item characteristic ‘discrimination’ (denoted *a*) is defined by the slope of the item characteristic curve (where the response probability is 0.5), reflecting the item’s ability to discriminate between respondents with high and low trait levels. Item *i* is more discriminative than item *j* (slope *a*_*i*_ is steeper than slope *a*_*j*_). The item characteristic ‘severity’ (denoted *b*) is defined by the trait level location where the response probability is 0.5. Item *j* is more severe than item *i* (*b*_*j*_ is greater than *b*_*i*_) because it takes (on average) a higher level of the trait to respond to item *j* than to item *i*.

According to the ‘graded response model’ [43], an item with 3 response categories is defined by 2 severity parameters and 1 discrimination parameter. The severity parameter is defined by the latent trait (theta) level above which the probability of endorsing a response category (or a higher category) is > 0.5. An item is said to ‘function’ the same across two groups when it demonstrates the same relationship with the underlying trait in these groups, that is, when the item has similar severity and discrimination parameters in both groups. DIF analysis, within the framework of IRT, thus involves testing the equivalence of the item parameters across two groups, controlling for the latent trait.

DIF analysis was performed by comparing each of the focal groups (adolescents and emerging adults) with the reference group (adults) in turn. We used the Wald test to assess the equivalence of item parameters, after linking the groups on a common latent trait scale. In the absence of pre-specified ‘anchor’ items, we followed a 3-stage procedure to first select appropriate anchor items and then testing items for DIF [44, 45]. At each stage a multi-group unidimensional IRT graded response model was fitted to each scale. At the first stage the item parameters were constrained to be the same across both groups to estimate the mean and variance of the latent trait of the focal group relative to the reference group. At the second stage the estimated latent means and variances were used to preliminarily link the groups, allowing all item parameters to be freely estimated and preliminarily tested for DIF. This stage was used to select items without DIF (p > 0.05) as anchor items. At the third stage the anchor items identified at stage 2 were used to link the groups, allowing means and variances to be freely estimated and the non-anchor items to be tested for DIF. Items with (Bonferroni corrected) p-values < 0.001 and unsigned item difference in the sample (UIDS) values (see next section) effect sizes > 0.1 were deemed to have DIF. A UIDS of 0.1 is comparable with a standardized mean difference in item score of 5% of the item range (which is 2 points in our case) [46].

To assess the magnitude of DIF, a final multi-group IRT model was fitted in which the parameters of the DIF-items were freely estimated while the parameters of the non-DIF items were constrained to be equal across the groups. The severity of DIF was then expressed as effect sizes based on expected item scores calculated twice based on either the item parameters of the reference group or the item parameters of the focal group [30]. The signed item difference in the sample (SIDS) represents the mean difference in expected item scores. The UIDS represents the mean of the absolute difference in expected item scores. Unlike the SIDS, the UIDS does not allow for cancellation of differences across respondents. The SIDS and UIDS are expressed in the metric of the scale score. In addition, we calculated the expected score standardized difference (ESSD), which is the Cohen’s *d* version of the SIDS (i.e. SIDS divided by the standard deviation of the item score). Absolute ESSD-values < 0.2 can be interpreted as a negligible effect, 0.2–0.5 as a small effect, 0.5–0.8 as a moderate effect, and > 0.8 as a large effect [47].

#### Differential test functioning

DIF at the item level may not have a great impact on the scale score, especially when separate items have oppositely directed DIF within a scale (this is called ‘DIF cancellation’) [27]. Therefore, we evaluated the scale-level impact of item-level DIF (i.e. differential test functioning (DTF) [48]) by calculating a number of effect size measures based on expected scale scores [30]. The signed test difference in the sample (STDS) is the sum of all SIDSs across the items of a scale, and allows for cancellation of differences in expected scores across items and persons. The unsigned test difference in the sample (UTDS) is the sum of all UIDSs across the items of a scale. The UTDS allows no cancellation across items or persons. The unsigned expected test score difference in the sample (UETSDS) is the average of absolute values of the expected test score differences in persons. As the UETSDS allows for cancellation across items but not across persons, this statistic reflects the true effect of DIF on observed scale scores. The expected test score standardized difference (ETSSD) is the Cohen’s *d* version of the STDS (i.e. STDS divided by the standard deviation of the scale score).

#### Software

The software package ‘lavaan’ version 06–2 [49], as implemented in the statistical program ‘R’ version 3.5.1 [50], was used for dimensionality analysis. The R-package ‘mirt’ version 1.26.3 was used to analyze DIF and DTF [51].

## Results

### Sample characteristics

The data comprised 1349 participants, about two thirds of them being female (Table 1). Given the mean 4DSQ scale scores, the adolescent group appeared to have lower symptom levels than the other groups.

**Table 1:** Participant characteristics by age group.

Characteristics	Age groups		
	Age 10–17	Age 18–25	Age 26–40
Numbers	243	358	748
Gender (% female)	63.8	69.3	63.9
4DSQ distress, range 0–32 (mean (SD))	14.6 (8.4)	18.7 (7.9)	19.6 (8.0)
4DSQ depression, range 0–12 (mean (SD))	3.3 (3.8)	3.9 (3.6)	3.5 (3.4)
4DSQ anxiety, range 0–24 (mean (SD))	5.1 (4.9)	6.0 (5.2)	5.3 (5.1)
4DSQ somatization, range 0–32 (mean (SD))	9.7 (6.2)	10.7 (6.3)	10.2 (6.4)

### Dimensionality

Most bifactor models (per scale, per age group) passed the criteria for adequate fit (Table 2). Only the RMSEA failed to reach its benchmark of < 0.06 in case of the distress scale in the adult group, and the depression scale in the adolescent and emerging adult groups. However, the other fit indices suggested adequate fit of these scales and there were no residual correlations > 0.2. In 2 of the somatization scale models a small number of absolute residual correlations > 0.2 could not be ameliorated by the bifactor model. All in all, we judged the bifactor models to fit the data reasonably well.

**Table 2:** Fit indices of the final bifactor models, by 4DSQ scale and age group; scaled statistics.

Scale/Age group		χ^2^	df	p	CFI	TLI	RMSEA	90% CI	SRMR	|resid| > 0.2
*Distress*										
	Age 10–17	58.42	47.3	0.129	0.993	0.997	0.031	0.000, 0.055	0.044	0.0%
	Age 18–25	100.9	51.4	0.000	0.977	0.990	0.052	0.037, 0.067	0.051	0.0%
	Age 26–40	228.8	58.5	0.000	0.969	0.989	0.062	0.053, 0.072	0.048	0.0%
*Depression*										
	Age 10–17	22.40	5.8	0.001	0.997	0.998	0.109	0.066, 0.155	0.046	0.0%
	Age 18–25	15.56	5.4	0.011	0.998	0.998	0.073	0.020, 0.129	0.031	0.0%
	Age 26–40	21.30	6.6	0.003	0.999	0.999	0.054	0.023, 0.089	0.024	0.0%
*Anxiety*										
	Age 10–17	36.36	28.1	0.137	0.992	0.994	0.035	0.000, 0.063	0.062	0.0%
	Age 18–25	59.96	33.7	0.004	0.982	0.989	0.047	0.025, 0.067	0.057	0.0%
	Age 26–40	67.66	38.8	0.003	0.993	0.995	0.032	0.017, 0.045	0.046	0.0%
*Somatization*										
	Age 10–17	48.03	40.0	0.180	0.990	0.991	0.029	0.000, 0.051	0.064	1.7%
	Age 18–25	92.96	44.9	0.000	0.955	0.966	0.055	0.041, 0.068	0.076	2.5%
	Age 26–40	100.0	55.0	0.000	0.982	0.988	0.033	0.023, 0.043	0.047	0.0%

df = degrees of freedom; CFI = comparative fit index; TLI = Tucker-Lewis index; RMSEA = root mean square error of approximation; 90% CI = 90% confidence interval of the RMSEA; SRMR = standardized root mean squared residual; |resid| = absolute residual correlation

Although the development of separate bifactor models in each age group, in principle, allowed for the appearance of different specific factors across the groups, this occurred only to a limited extent (see S1 File for the factor loadings). The distress scale demonstrated two solid and reproducible specific factors and two weaker and more variable specific factors. The solid specific factors comprised of only two items each, providing additional information about sleeping problems (items #20 and #39) and traumatic experiences (items #47 and #48) over and above the information conveyed by the general factor. The PUC was > 0.8, suggesting essential unidimensionality of the distress scale across the age groups (Table 3).

**Table 3:** Dimensionality statistics of the final bifactor models, by 4DSQ scale and age group.

Scale/Age group		PUC	ECV	Omega-h
*Distress*				
	Age 10–17	0.850	0.698	0.878
	Age 18–25	0.925	0.656	0.877
	Age 26–40	0.950	0.737	0.918
*Depression*				
	Age 10–17	0.800	0.904	0.918
	Age 18–25	0.933	0.904	0.914
	Age 26–40	0.933	0.932	0.931
*Anxiety*				
	Age 10–17	0.894	0.803	0.870
	Age 18–25	0.939	0.852	0.888
	Age 26–40	0.955	0.928	0.915
*Somatization*				
	Age 10–17	0.892	0.563	0.803
	Age 18–25	0.908	0.572	0.803
	Age 26–40	0.892	0.574	0.803

PUC = proportion of uncontaminated correlations; ECV = explained common variance; Omega-h = omega-hierarchical

The depression scale showed one fairly reproducible, but nevertheless rather weak, specific factor revolving around suicidal ideation (items #33 and #46). The PUC was > 0.8 suggesting essential unidimensionality. The anxiety scale demonstrated two rather weak specific factors that were each reproduced in only two age groups. A specific social anxiety factor (items #23 and #44) appeared in the adolescent and emerging adult groups, but not in the adult group, whereas a specific free floating anxiety factor (items #21 and #27) appeared in the emerging adult and adult groups but not in the adolescent group. The PUC values were > 0.8 suggesting essential unidimensionality.

The somatization scale demonstrated three substantively solid and fairly reproducible specific factors, corresponding to well-known clusters of unexplained physical symptoms: cardiovascular (items #15 and #16), gastro-intestinal (items #9, #12 and #13) and musculoskeletal symptoms (items #2, #4 and #5) [52, 53]. A weaker fourth specific factor appeared in the emerging adult and adult groups but was not reproduced in the adolescent group. The PUC values were again > 0.8 suggesting essential unidimensionality.

### Differential item functioning (DIF)

**Fig 2:**
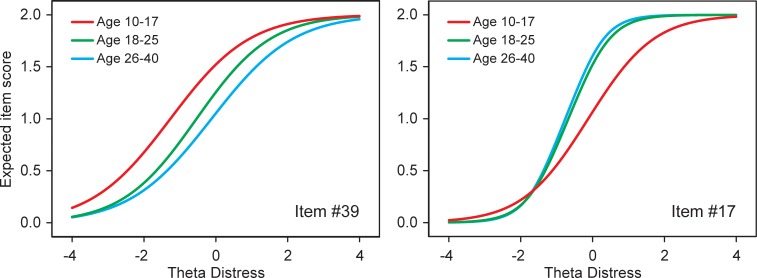
Two selected items with differential item functioning. Expected item scores as a function of the DIF-free latent trait (theta), by age group. Item #39: ‘difficulty getting to sleep’. Item #17: ‘feeling down or depressed’.

DIF was revealed in 9 items across the adolescent and adult groups, and in 4 items across the emerging adult and adult groups (Table 4). The DIF was mostly small or moderate in terms of effect size (ESSD), except for item #39 (‘difficulty getting to sleep’) for which the effect size was large. The SIDS of 0.432 for item #39 means that, conditional on the latent distress trait, adolescents scored on average 0.432 points higher for that item than adults (the item scale is 2 points). The ESSD of 1.148 means that 0.432 is 1.148 standard deviation of the item score, a large effect size. Fig 2 (left panel) displays the expected item score of item #39 as a function of the (DIF-free) trait score (theta) for distress. The figure demonstrates that item #39 was uniformly less severe for adolescents than for adults: at each level of distress, adolescents more often experienced problems falling asleep, resulting in higher expected item scores for this age group. Apparently, adolescents have lower thresholds for problems falling asleep than adults. Potentially, this could have been translated into an increase of the (average) distress scale score for adolescents by 0.432 points (SIDS-value), not accounted for by increased true levels of distress.

**Table 4:** Items with differential item functioning (DIF) by age group. Entries are effect sizes (reference group: adults, age 26–40). Empty cells indicate absence of DIF in the relevant comparison.

			Adolescents (age 10–17)			Emerging adults (age 18–25)		
Scale	Item	Short item description	SIDS	UIDS	ESSD	SIDS	UIDS	ESSD
Distress	#17	feeling down or depressed	-0.292	0.307	-0.548			
	#19	worry	-0.217	0.217	-0.452			
	#25	feeling tense	-0.210	0.211	-0.403			
	#37	inability to do anything	0.200	0.208	0.335	0.129	0.138	0.215
	#39	difficulty getting to sleep	0.432	0.432	1.148			
Depression	#34	inability to enjoy anything	-0.286	0.286	-0.476	-0.167	0.167	-0.308
Anxiety	#42	specific phobia	0.152	0.155	0.443	0.122	0.139	0.335
	#43	fear of public transport				0.094	0.101	0.399
Somatization	#4	neck pain	-0.284	0.284	-0.785			
	#10	blurred vision	0.182	0.190	0.544			

SIDS = signed item difference in the sample; UIDS = unsigned item difference in the sample; ESSD = expected score standardized difference

On the other hand, however, other distress items turned out to be more severe for adolescents, for example item #17 (‘feeling down or depressed’). The SIDS of -0.292 for this items means that, conditional on the latent distress trait, adolescents scored on average 0.292 points lower for that item than adults. The ESSD of -0.548 means that -0.292 is -0.548 standard deviation of the item score, a moderate effect size. The expected item score of item #17, as a function of the latent trait, is shown in Fig 2 (right panel). Going from minimal to maximal distress the expected item score of item #17 for adolescents was increasingly lagging behind the expected item score for adults (and emerging adults). Note that the slope of the expected item score curve is related to the slope of the item characteristic curve (see Fig 1), and thus reflects the item’s discriminative ability. The item parameters from the final multi-group IRT models (see S2 File) shows that the *a* parameter of item #17 was 1.199 for adolescents and 2.068 and 2.310 for the emerging adults and adults, respectively. The more gradual slope of item #17 for adolescents can be interpreted as a smaller correlation between the item and the distress trait, compared with the (emerging) adult groups.

The functioning of the DIF-laden items in emerging adults was, in most cases, intermediate between adolescents and adults (see S2 File).

### Differential test functioning (DTF)

**Fig 3:**
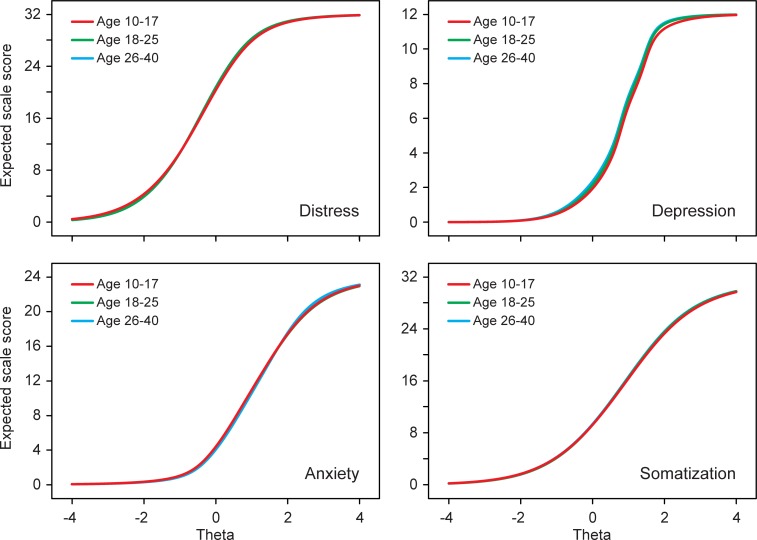
Test characteristic curves. Expected scale scores as a function of the DIF-free latent trait (theta).

The impact of DIF at scale level was small (Table 5) and in terms of effect size (ETSSD) negligible. This was, at least partly, due to DIF cancellation, which means that the effect of more severe items was cancelled out by the opposite effect of less severe items within the same scale. The UTDS-value of 1.375 for distress means that if the DIF in the distress items would have worked in the same direction, adolescents would have mean distress scores 1.375 points higher (or lower) than adults with similar true levels of distress. However, because the direction of DIF varied across the items (3 items were more severe and 2 items were less severe) the mean difference in expected distress score was only -0.088. Fig 3 shows the expected scale scores as a function of the DIF-free trait, demonstrating that the scale scores functioned virtually the same across the age groups.

**Table 5:** Differential test functioning (DTF) by age group. Entries are effect sizes (reference group: adults, age 26–40). Empty cells indicate absence of DTF in the relevant comparison.

	Adolescents (age 10–17)				Emerging adults (age 18–25)			
Scale	STDS	UTDS	UETSDS	ETSSD	STDS	UTDS	UETSDS	ETSSD
Distress	-0.088	1.375	0.185	-0.011	0.129	0.138	0.138	0.018
Depression	-0.286	0.286	0.286	-0.076	-0.167	0.167	0.167	-0.050
Anxiety	0.152	0.155	0.155	0.035	0.216	0.239	0.234	0.047
Somatization	-0.102	0.474	0.102	-0.019				

STDS = signed test difference in the sample; UTDS = unsigned test difference in the sample; UETSDS = unsigned expected test score difference in the sample; ETSSD = expected test score standardized difference

## Discussion

### Main findings

The present study investigated whether adolescents (age 10–17) and emerging adults (age 18–25) respond to the items of the 4DSQ in the same way as adults (age 26–40). This is an important requisite in order to determine whether using the 4DSQ is justified in adolescents and emerging adults and whether their 4DSQ scores can be interpreted in the same way as in adults. Adolescents responded slightly but significantly differently to 9 items, and emerging adults to 4 items out of a total of 50 items, when compared to adults. The thresholds of 5 of the DIF items (#10, #37, #39, #42 and #43) were lower compared to the adult reference group, suggesting that these items represented less severe symptoms for the focal group(s). The thresholds of 5 other DIF items (#4, #17, #19, #25 and #34) were higher compared to the adult reference group, indicating that these items were more severe. The degree of DIF in emerging adults was generally somewhat smaller than in adolescents, suggesting a developmental process by which deviating scoring patterns in adolescents (compared to adults) gradually ‘normalized’ over the years. However, due to DIF cancellation, these deviant scoring patterns in adolescents and emerging adults did not result in clinically relevant DTF, suggesting that the scale scores were essentially comparable.

The question may be raised as to whether, if adolescents report more sleep problems but fewer low mood and worries, their distress scores are really comparable to adults. Indeed, distress in adolescents might *look* slightly different from distress in adults (i.e., more sleep problems, less low mood and worries). However, symptoms do not constitute the construct distress. Rather, symptoms are an expression of the construct distress, but due to DIF, the same level of distress may cause slightly different symptoms in adolescents than in adults. However, due to DIF cancellation, the same level of distress causes comparable distress scale scores across the age groups. As a consequence, the same distress scale scores indicate comparable levels of distress across the groups, despite slightly different symptom patterns underlying these scores.

In summary, the distress scale score functions the same across the groups as indicator of the underlying latent distress trait. The same applies to the other 4DSQ scales. Therefore, 4DSQ scores from adolescents and emerging adults can safely be interpreted in the same way as from adults. Consequently, 4DSQ scores might meaningfully be followed-up and compared in longitudinal studies across adolescence and emerging adulthood, and into the adult years.

### Possible causes of DIF

The item with the largest degree of DIF (i.e. ‘difficulty getting to sleep’, item #39) demonstrated a reduced threshold for adolescents. Adolescence is known for its shift of the circadian rhythm [54], rendering adolescents vulnerable to problems with sleep initiation. Social factors and technology (e.g., smartphone) use may further aggravate the problem. Trouble falling asleep is among the most frequently reported sleep problems in adolescents [55]. Whereas the similar slopes of the expected item score curves (see Fig 2) indicate that difficulty getting to sleep is equally related to distress in adolescents and adults, due to their increased ‘baseline’ vulnerability, difficulty getting to sleep occurs in adolescents at lower distress levels, compared to adults. Interestingly, another sleep complaint (i.e. ‘disturbed sleep’, item #20) functioned the same in adolescents and adults.

Adolescents also experienced a reduced threshold for ‘inability to do anything’ (item #37), which may also be related to the circadian shift mentioned above. On the other hand, the thresholds for ‘feeling down or depressed’ (item #17), ‘worry’ (item #19), and ‘feeling tense’ (item #25) appeared to be increased in adolescents. Apparently, compared to adults, adolescents are more resistant against low mood, less inclined to engage in worry, and they stay relatively more relaxed when distressed. However, regarding the assumed resilience to low mood, a different type of explanation may be offered: the Dutch 4DSQ employs a particular Dutch expression in item #17, namely ‘neerslachtigheid’ (English: dejection, gloom, dreariness, dismalness). This word is a little old-fashioned and adolescents may not be as familiar with its meaning as emerging adults and adults. Consequently, some adolescents may not recognize ‘neerslachtigheid’ as an accurate description of their low mood. As a result, ‘neerslachtigheid’ is to a slightly lesser extent a characteristic of distress in adolescents than it is in (emerging) adults, hence the reduced item-trait correlation.

### Limitations and strengths

Strengths of this study include the relatively large sample sizes, the use of state-of-the-art statistical methods to analyze DIF and DTF, and the distinction between emerging adults and adults to obtain a more detailed picture of the differences between adolescence and adulthood.

The main limitation of the present study is that data were obtained from a single psychotherapy practice. This may limit the generalizability of the results both to other practices in other parts of the country, and to other settings (e.g. family practice). Furthermore, the results may not be applicable to the general population. On the other hand, the fact that adolescent and (emerging) adult data were collected in the same practice/setting may also represent a strength because this ensures that the difference between the groups can only be traced back to age (and not to e.g. differences in urbanization or socioeconomic family status). A second limitation is that the results may not be generalizable to other language versions of the 4DSQ. The supposed cause of DIF in item #17 is probably specific to the Dutch language.

## Conclusion

Adolescents and emerging adults score the 4DSQ items not exactly in the same way as adults do, but this has no effect on the scale scores. Thus, practically speaking, the 4DSQ scales measure the same constructs (i.e. distress, depression, anxiety and somatization) in adolescents and emerging adults in the same way as they do in adults.

## Supporting information

S1 File Factor loadings of the unidimensional and bifactor models. (PDF)

S2 File Item parameters by age group. (PDF)

S3 File Dataset (plain text file). (TXT)
