# Supplementary material for: Measurement equivalence of the Four-Dimensional Symptom Questionnaire (4DSQ) in adolescents and emerging adults
Source: PLoS One. 2019 Aug 29;14(8):e0221904. doi: 10.1371/journal.pone.0221904 (PMC6715201; doi:10.1371/journal.pone.0221904)
Supplement: S1 File — (PDF) [file pone.0221904.s001.pdf]

Supporting Information 1

Factor loadings of the unidimensional and bifactor models

Article title: Measurement equivalence of the Four-Dimensional Symptom Questionnaire (4DSQ) in adolescents and emerging adults

Authors: Berend Terluin, Johannes C. van der Wouden, Henrica C. W. de Vet

Explanation

The tables present standardized factor loadings of the unidimensional models and the bifactor models per 4DSQ scale per age group. In addition, the following bifactor statistics are presented: omega-total (omega-t), omega-hierarchical (omega-h), omega-subgroup (omega-s) and explained common variance (ECV). The omega-coefficients are based on a partitioning of the test-level (scale score) variance. Omega-t represents the test-level variance accounted for by all factors, divided by the total test-level variance (including error variance). As a reliability estimate, omega-t is comparable with Cronbach's alpha. Omega-t can also be determined for subsets of items making up specific factors. Omega-h represents the test-level variance accounted for by the general factor, divided by the total test-level variance. Omega-s represents the test-level variance of a subset of items making up a specific factor (i.e. subscale), accounted for by the specific factor, divided by the total variance of the subset of items. Omega-s thus is the proportion of reliable test-level variance that a subscale adds over and beyond the variance accounted for by the general factor. The ECV represents the proportion of the total reliable item-level variance that is accounted for by the respective factors.

The bifactor model of distress in 10-17 year old adolescents (see below) show that 69.8% of the total reliable item-level variance is accounted for by the general factor (ECV). The specific factors account for much less item-level variance: 8.3%, 7.5%, 2.9% and 11.4% respectively. Omega-t of the distress scale (16 items) indicates that the proportion reliable test-level variance (accounted for by 5 factors) is 0.96. The general factor alone accounts for 87.8% of the total test-level variance (omega-h). If we would use a subscale of items #20 and #39 (making up specific factor F1), this subscale would have a reliable proportion of test-level variance of 0.885 (omega-t of subscale F1). Part of this reliable variance is accounted for by the general factor, but nevertheless 50.1% of the variance is explained by the items making up F1 over and beyond the general factor (omega-s). The omega-s values indicate that F1 and F2 do contribute some reliable variance to what is already captured by the general factor, but this cannot be said of F3 and F4.

**Distress Age 10-17**		**Unidim. model**	**Bifactor model**				
	**Item**		**Gen**	**F1**	**F2**	**F3**	**F4**
	DSQ17	0.617	0.658				
	DSQ19	0.727	0.745			0.393	
	DSQ20	0.740	0.678	0.667			
	DSQ22	0.763	0.722				0.291
	DSQ25	0.695	0.710			0.393	
	DSQ26	0.640	0.684				
	DSQ29	0.830	0.739				0.445
	DSQ31	0.690	0.568				0.537
	DSQ32	0.828	0.715				0.508
	DSQ36	0.832	0.761				0.381
	DSQ37	0.783	0.672				0.494
	DSQ38	0.688	0.734				
	DSQ39	0.606	0.491	0.667			
	DSQ41	0.728	0.777				
	DSQ47	0.650	0.582		0.633		
	DSQ48	0.677	0.617		0.633		
Omega-t			0.960	0.885	0.864	0.812	0.930
Omega-h			0.878				
Omega-s				0.501	0.455	0.183	0.268
ECV			0.698	0.083	0.075	0.029	0.114

**Distress Age 18-25**		**Unidim. model**	**Bifactor model**				
	**Item**		**Gen**	**F1**	**F2**	**F3**	**F4**
	DSQ17	0.720	0.765				
	DSQ19	0.724	0.758				
	DSQ20	0.742	0.605	0.722			
	DSQ22	0.728	0.698			0.350	
	DSQ25	0.750	0.792				
	DSQ26	0.565	0.601				
	DSQ29	0.713	0.676			0.388	
	DSQ31	0.679	0.622			0.459	
	DSQ32	0.817	0.804				0.400
	DSQ36	0.810	0.796				0.400
	DSQ37	0.758	0.694			0.550	
	DSQ38	0.630	0.666				
	DSQ39	0.666	0.489	0.722			
	DSQ41	0.649	0.685				
	DSQ47	0.628	0.400		0.798		
	DSQ48	0.667	0.460		0.798		
Omega-t			0.957	0.903	0.902	0.909	0.906
Omega-h			0.877				
Omega-s				0.566	0.695	0.262	0.236
ECV			0.656	0.096	0.118	0.091	0.039

Distress<br>Age 26-40		Unidim.<br>model	Bifactor model				
	**Item**		**Gen**	**F1**	**F2**	**F3**	**F4**
	DSQ17	0.774	0.800				
	DSQ19	0.754	0.775				
	DSQ20	0.656	0.604	0.678			
	DSQ22	0.805	0.832				
	DSQ25	0.742	0.765				
	DSQ26	0.600	0.624				
	DSQ29	0.831	0.812			0.282	
	DSQ31	0.758	0.735			0.338	
	DSQ32	0.828	0.788				0.450
	DSQ36	0.816	0.777				0.450
	DSQ37	0.868	0.841			0.411	
	DSQ38	0.695	0.720				
	DSQ39	0.557	0.483	0.678			
	DSQ41	0.649	0.671				
	DSQ47	0.632	0.425		0.773		
	DSQ48	0.699	0.529		0.773		
Omega-t			0.963	0.862	0.905	0.902	0.898
Omega-h			0.918				
Omega-s				0.525	0.656	0.142	0.223
ECV			0.737	0.084	0.109	0.033	0.037

**Depression Age 10-17**		**Unidim. model**	**Bifactor model**	
	**Item**		**Gen**	**F1**
	DSQ28	0.839	0.893	
	DSQ30	0.978	0.893	0.401
	DSQ33	0.978	0.893	0.401
	DSQ34	0.785	0.820	
	DSQ35	0.785	0.820	
	DSQ46	0.978	0.893	0.401
Omega-t			0.967	0.986
Omega-h			0.918	
Omega-s				0.166
ECV			0.904	0.096

**Depression Age 18-25**		**Unidim. model**	**Bifactor model**	
	**Item**		**Gen**	**F1**
	DSQ28	0.813	0.812	
	DSQ30	0.918	0.992	
	DSQ33	0.975	0.872	0.468
	DSQ34	0.745	0.763	
	DSQ35	0.658	0.672	
	DSQ46	0.958	0.836	0.468
Omega-t			0.947	0.974
Omega-h			0.914	
Omega-s				0.225
ECV			0.904	0.096

**Depression Age 26-40**		**Unidim. model**	**Bifactor model**	
	**Item**		**Gen**	**F1**
	DSQ28	0.833	0.837	
	DSQ30	0.925	0.980	
	DSQ33	0.990	0.897	0.402
	DSQ34	0.767	0.775	
	DSQ35	0.714	0.726	
	DSQ46	0.969	0.897	0.402
Omega-t			0.954	0.983
Omega-h			0.931	
Omega-s				0.164
ECV			0.932	0.068

**Anxiety Age 10-17**	**Item**	**Unidim. model**	**Bifactor model Gen**	**F1**	**F2**
	DSQ18	0.704	0.664		0.341
	DSQ21	0.879	0.887		
	DSQ23	0.616	0.594	0.522	
	DSQ24	0.795	0.807		
	DSQ27	0.909	0.918		
	DSQ40	0.719	0.733		
	DSQ42	0.602	0.613		
	DSQ43	0.620	0.634		
	DSQ44	0.493	0.461	0.522	
	DSQ45	0.758	0.698		0.434
	DSQ49	0.683	0.608		0.521
	DSQ50	0.379	0.296		0.493
Omega-t			0.929	0.713	0.823
Omega-h			0.870		
Omega-s				0.353	0.316
ECV			0.803	0.079	0.118

**Anxiety Age 18-25**	**Item**	**Unidim. model**	**Bifactor model Gen**	**F1**	**F2**
	DSQ18	0.637	0.656		
	DSQ21	0.870	0.778		0.433
	DSQ23	0.633	0.598	0.507	
	DSQ24	0.772	0.807		
	DSQ27	0.922	0.848		0.433
	DSQ40	0.695	0.718		
	DSQ42	0.567	0.583		
	DSQ43	0.635	0.651		
	DSQ44	0.500	0.459	0.523	
	DSQ45	0.730	0.752		
	DSQ49	0.692	0.689	0.203	
	DSQ50	0.366	0.377		
Omega-t			0.920	0.768	0.919
Omega-h			0.888		
Omega-s				0.255	0.203
ECV			0.852	0.090	0.059

Anxiety<br>Age 26-40		Unidim.<br>model	Bifactor model	
	**Item**		**Gen**	**F1**
	DSQ18	0.644	0.664	
	DSQ21	0.929	0.852	0.352
	DSQ23	0.613	0.631	
	DSQ24	0.821	0.775	0.302
	DSQ27	0.880	0.779	0.516
	DSQ40	0.786	0.806	
	DSQ42	0.722	0.743	
	DSQ43	0.780	0.792	
	DSQ44	0.499	0.514	
	DSQ45	0.683	0.701	
	DSQ49	0.744	0.766	
	DSQ50	0.470	0.481	
Omega-t			0.932	0.924
Omega-h			0.915	
Omega-s				0.177
ECV			0.928	0.072

Somatization<br>Age 10-17		Unidim.<br>model	Bifactor model				
	**Item**		**Gen**	**F1**	**F2**	**F3**	**F4**
	DSQ01	0.680	0.754				
	DSQ02	0.410	0.385			0.337	
	DSQ03	0.375	0.435				
	DSQ04	0.603	0.511			0.582	
	DSQ05	0.603	0.511			0.730	
	DSQ06	0.451	0.453				0.327
	DSQ07	0.612	0.629				0.585
	DSQ08	0.608	0.606		0.214		
	DSQ09	0.571	0.551		0.264		
	DSQ10	0.463	0.519				
	DSQ11	0.643	0.708				
	DSQ12	0.695	0.478		0.691		
	DSQ13	0.661	0.415		0.825		
	DSQ14	0.231	0.173				0.596
	DSQ15	0.815	0.722	0.590			
	DSQ16	0.780	0.652	0.590			
Omega-t			0.918	0.901	0.832	0.777	0.711
Omega-h			0.803				
Omega-s				0.382	0.404	0.450	0.420
ECV			0.563	0.081	0.148	0.114	0.093

**Somatization Age 18-25**	**Unidim. model**	**Bifactor model**				
**Item**		**Gen**	**F1**	**F2**	**F3**	**F4**
DSQ01	0.555	0.585				0.442
DSQ02	0.532	0.385			0.586	
DSQ03	0.361	0.413				
DSQ04	0.637	0.451			0.734	
DSQ05	0.586	0.411			0.657	
DSQ06	0.456	0.508				
DSQ07	0.490	0.556				
DSQ08	0.505	0.489			0.256	
DSQ09	0.694	0.590		0.500		
DSQ10	0.554	0.576				0.442
DSQ11	0.567	0.636				
DSQ12	0.679	0.564		0.545		
DSQ13	0.704	0.541		0.707		
DSQ14	0.549	0.610				
DSQ15	0.673	0.618	0.592			
DSQ16	0.709	0.643	0.592			
Omega-t		0.915	0.856	0.857	0.811	0.694
Omega-h		0.803				
Omega-s			0.402	0.443	0.506	0.255
ECV		0.572	0.085	0.127	0.168	0.047

**Somatization Age 26-40**	**Unidim. model**	**Bifactor model**				
**Item**		**Gen**	**F1**	**F2**	**F3**	**F4**
DSQ01	0.673	0.722				0.320
DSQ02	0.553	0.448			0.689	
DSQ03	0.441	0.495				
DSQ04	0.550	0.464			0.570	
DSQ05	0.511	0.418			0.567	
DSQ06	0.471	0.521				
DSQ07	0.621	0.597	0.309			
DSQ08	0.538	0.590				
DSQ09	0.554	0.464		0.488		
DSQ10	0.647	0.684				0.320
DSQ11	0.663	0.605	0.391			
DSQ12	0.664	0.500		0.688		
DSQ13	0.657	0.477		0.708		
DSQ14	0.547	0.602				
DSQ15	0.713	0.577	0.770			
DSQ16	0.664	0.556	0.487			
Omega-t		0.921	0.856	0.837	0.798	0.747
Omega-h		0.803				
Omega-s			0.353	0.528	0.522	0.128
ECV		0.574	0.127	0.143	0.132	0.024
