# Supplementary material for: Measurement equivalence of the Four-Dimensional Symptom Questionnaire (4DSQ) in adolescents and emerging adults
Source: PLoS One. 2019 Aug 29;14(8):e0221904. doi: 10.1371/journal.pone.0221904 (PMC6715201; doi:10.1371/journal.pone.0221904)
Supplement: S2 File — (PDF) [file pone.0221904.s002.pdf]

Supporting Information 2

Item parameters by age group (items with DIF highlighted)

Article title: Measurement equivalence of the Four-Dimensional Symptom Questionnaire (4DSQ) in adolescents and emerging adults
Authors: Berend Terluin, Johannes C. van der Wouden, Henrica C. W. de Vet

**Scale/Items**	**Adolescents (age 10-17)**			**Emerging adults (age 18-25)**			**Young adults (age 26-40)**		
**Distress**	***a***	***b1***	***b2***	***a***	***b1***	***b2***	***a***	***b1***	***b2***
#17	1.199	-0.652	0.411	2.068	-1.139	-0.210	2.310	-1.236	-0.285
#19	1.690	-1.799	-0.910	2.123	-2.106	-1.138	2.138	-2.396	-1.222
#20	1.352	-1.611	-0.389	1.352	-1.611	-0.389	1.352	-1.611	-0.389
#22	2.304	-0.876	0.008	2.304	-0.876	0.008	2.304	-0.876	0.008
#25	1.495	-1.476	-0.275	2.199	-1.467	-0.509	2.046	-1.782	-0.706
#26	1.336	-1.923	-0.365	1.336	-1.923	-0.365	1.336	-1.923	-0.365
#29	2.507	-0.503	0.317	2.507	-0.503	0.317	2.507	-0.503	0.317
#31	1.925	-0.408	0.707	1.925	-0.408	0.707	1.925	-0.408	0.707
#32	2.466	-0.438	0.446	2.466	-0.438	0.446	2.466	-0.438	0.446
#36	2.467	-0.431	0.538	2.467	-0.431	0.538	2.467	-0.431	0.538
#37	2.087	-1.029	0.178	2.284	-0.818	0.187	3.106	-0.600	0.351
#38	1.607	-0.871	0.352	1.607	-0.871	0.352	1.607	-0.871	0.352
#39	0.968	-1.806	-0.724	1.036	-1.046	-0.027	0.951	-0.734	0.494
#41	1.548	-1.344	-0.374	1.548	-1.344	-0.374	1.548	-1.344	-0.374
#47	0.860	-0.921	0.526	0.860	-0.921	0.526	0.860	-0.921	0.526
#48	1.046	-0.522	0.591	1.046	-0.522	0.591	1.046	-0.522	0.591
**Depression**									
#28	2.177	-0.193	0.715	2.177	-0.193	0.715	2.177	-0.193	0.715
#30	4.423	0.468	1.193	4.423	0.468	1.193	4.423	0.468	1.193
#33	9.772	0.777	1.473	9.772	0.777	1.473	9.772	0.777	1.473
#34	1.548	0.413	1.560	1.872	-0.012	1.029	1.970	-0.285	0.700
#35	1.571	0.072	1.168	1.571	0.072	1.168	1.571	0.072	1.168
#46	5.898	0.859	1.518	5.898	0.859	1.518	5.898	0.859	1.518

**Anxiety**									
#18	1.442	0.434	1.453	1.442	0.434	1.453	1.442	0.434	1.453
#21	3.694	-0.252	0.350	3.694	-0.252	0.350	3.694	-0.252	0.350
#23	1.318	1.156	2.128	1.318	1.156	2.128	1.318	1.156	2.128
#24	2.483	0.516	1.196	2.483	0.516	1.196	2.483	0.516	1.196
#27	3.445	-0.155	0.573	3.445	-0.155	0.573	3.445	-0.155	0.573
#40	1.972	1.233	1.810	1.972	1.233	1.810	1.972	1.233	1.810
#42	1.288	0.704	1.548	1.200	0.898	1.699	1.873	1.007	1.600
#43	1.630	1.763	2.431	1.434	1.783	2.279	2.509	1.809	2.098
#44	0.890	0.412	1.766	0.890	0.412	1.766	0.890	0.412	1.766
#45	1.858	1.277	1.922	1.858	1.277	1.922	1.858	1.277	1.922
#49	1.813	1.182	1.819	1.813	1.182	1.819	1.813	1.182	1.819
#50	0.773	1.715	3.223	0.773	1.715	3.223	0.773	1.715	3.223
**Somatization**									
#01	1.564	-0.185	1.152	1.564	-0.185	1.152	1.564	-0.185	1.152
#02	0.936	-0.405	1.123	0.936	-0.405	1.123	0.936	-0.405	1.123
#03	1.127	3.706	6.341	1.127	3.706	6.341	1.127	3.706	6.341
#04	1.175	0.266	1.497	1.207	-0.060	0.938	1.029	-0.312	0.740
#05	0.999	-0.386	0.979	0.999	-0.386	0.979	0.999	-0.386	0.979
#06	0.945	0.152	1.516	0.945	0.152	1.516	0.945	0.152	1.516
#07	1.318	0.450	1.588	1.318	0.450	1.588	1.318	0.450	1.588
#08	1.141	-0.980	0.516	1.141	-0.980	0.516	1.141	-0.980	0.516
#09	1.145	-0.006	1.183	1.145	-0.006	1.183	1.145	-0.006	1.183
#10	0.916	0.107	1.854	1.319	0.401	1.930	1.469	0.543	1.674
#11	1.470	0.547	1.541	1.470	0.547	1.541	1.470	0.547	1.541
#12	1.248	-0.294	0.922	1.248	-0.294	0.922	1.248	-0.294	0.922
#13	1.191	0.229	1.384	1.191	0.229	1.384	1.191	0.229	1.384
#14	1.065	1.167	2.144	1.065	1.167	2.144	1.065	1.167	2.144
#15	1.593	0.629	1.599	1.593	0.629	1.599	1.593	0.629	1.599
#16	1.590	1.230	2.108	1.590	1.230	2.108	1.590	1.230	2.108
